# Supplementary material for: Advances in Novel Animal Vitamin C Biosynthesis Pathways and the Role of Prokaryote-Based Inferences to Understand Their Origin
Source: Genes (Basel). 2022 Oct 21;13(10):1917. doi: 10.3390/genes13101917 (PMC9602106; doi:10.3390/genes13101917)
Supplement: Supplementary file 1 [file genes-13-01917-s001.zip › genes-1954231-supplementary.pdf]

Advances in Novel Animal Vitamin C Biosynthesis Pathways and the Role of Prokaryote-Based Inferences to Understand Their Origin

Pedro Duque, Cristina P. Vieira, Jorge Vieira

Supplementary Figures

Figure S1 – Complete AO dataset MUSCLE alignment file. The alignment presented was the one used to perform the phylogenetic analyses discussed in the manuscript. The relevant amino acid regions used to extrapolate putative AO functions are tagged at the top of the respective alignment positions, and delimited by the black colour background.

	1 10 20 30 40 50 60 70 80 90
Mycobacterium tuberculosis H37Rv NP_216287.1	--
Streptomyces albiflaviniger MBI0379321.1	---MAV
Pseudomonas aeruginosa PAO1 NP_254014.1	---MVGNP
Myxococcales bacterium MCO4763851.1	--
Bacteroidetes bacterium MBS1617249.1	--
Flavobacteriales bacterium HIA12326.1	--
Moorena sp. SIO4G3 NEO81287.1	--
Calothrix parasitica NIES 267 BAY85903.1	--
Asgard group archaeon MCP8715902.1	--
Halobacteriales archaeon PSP48796.1	--
Euglena gracilis BAO94255.2	MARNKVMRMLF----------RAVA------------------------------------PTTAGICLWQVQCNFKSSE-----VEVWC
Trypanosoma brucei brucei TREU927 XP_844937.1	---M
Leishmania major strain Friedlin XP_001682394.1	--
Mus musculus NP_848862.1	--
Rattus norvegicus EDL85374.1	--
Xenopus laevis OCT81467.1	--
Ciona intestinalis XP_002122023.1	---MTS
Falco cherrug XP_005443605.1	-MGPAGKGAAD----------TPGR------------------------------------ALSASLEKFGQFGAKVSLW-----LEAEA
Rosa chinensis XP_024182616.1	MQRALTVKRSL-QSL------QRIANSTTK---NPLSSGRAFC----NAPSPSPSPAPSASSASASASASEVRKYMGYTA-----LVLFC
Rosa chinensis XP_024165881.1	---MYRSWWLLGAIH--------LLVSV
Prunus mume XP_008240731.1	MLRALTLRRSL-QSLHHNHLKPLISTAIPKNPLNPLSPARACS----SLSSPSAS-----------SSASELRKYLGYTA-----LVLFS
Prunus mume XP_008220057.1	---MHSFWWLLCALHHVIWAA--TMLIS
Fragaria vesca subsp vesca XP_004291192.1	---MYSSWWLLGVL---------HLLVS
Fragaria vesca subsp vesca XP_004303609.1	MQRALTLKRTL-QSL------PRI----TK---NPLIPGRAFC----NASTPSPSP----------ASASELRKYMGYTA-----LVLFC
Arabidopsis thaliana NP_190376.1	MLRSLLLRRSVGHSL------GTLSPSSST-IRSSFSPHRTLCTTGQTLTPPPPPPPRPPPPPPATASEAQFRKYAGYAA-----LAIFS
Arabidopsis thaliana NP_182199.1	---MAFTSSPSYGSLNAAFWRTIFVVHCIS-----TLVFT
Arabidopsis thaliana NP_564393.1	--MAFWLSLI-FFCFC-----TF---
Arabidopsis thaliana NP_182198.2	---MAFTFPPSYRTLVGLYYIF-----TLMHT
Arabidopsis thaliana NP_182197.1	---MAFGYSPSYCSFWRTLLGLYCLF-----TLVHT
Arabidopsis thaliana NP_200460.1	---MSFWLSLI--FCFF------TF---
Quercus suber XP_023896433.1	---MVLLRGVFQFSCFF------LLIFG
Quercus suber XP_023896434.1	---MRNINVNQLRCIFNTKKKAPPKLLNMVLLRGLFQSCCLF-----LLIFV
Quercus suber XP_023900169.1	MLRALVLKRSHPSSLHHHHLLKPLTSTTAT-------TTRPFC----T-TAPTPTPIAS-------STTSELRKYLGYTA-----LVLFC
Quercus suber XP_023914113.1	---MYTTWNWFLGVLHLVTWGSSSTLLT
Aspergillus chevalieri XP_043131130.1	--MVGLKL-----QLLLV
Candida dubliniensis CD36 XP_002422445.1	--
Saccharomyces cerevisiae S288C NP_013624.1	--
Thermothielavioides terrestris XP_003651626.1	---M
Colletotrichum lupini XP_049145240.1	--
Homo sapiens DHCR24 NP_055577.1	-------MEPA----------VSLA------------------------------------VCALLFLLWVRLKGLEFVLIHQR-WVFVC
Bombyx mori DHCR24 XP_004926865.1	---MAIETETFLEYLVVEYR-------WVIVI

FAD-Binding motif
(X[GR]X[GS][HLK][SG]) region

	91 100 110 120 130 140 150 160 170 180 190 200 210 215
NP_216287.1	------MSPI-----------------------WSNW--PGEQVCA-PSAIVRPTSEAELADVIAQAAKRGERVRAVGS-GHSFTDI-ACTD-------GVMIDMTGLQRVLDVDQ---------
MBI0379321.1	RTVKTVRSSV-----------------------WRNW--AGNVMAR-PTRIVAPSSTQELAEMVRRATAEGLKVKAVGT-GHSFTTA-AATD-------GLLIRPDRMAGVRGLDR---------
NP_254014.1	ALAELLRAPRLIP--------------------WRNW--SGAQSCL-PLAREAPKDLDELVRIIRQA---SGRIRPVGS-GHSFSAL-VPTD-------GTLLSLAYFSGLLSHDS---------
MCO4763851.1	-------MSQS----------------------IANW--SRTHHCT-PARLERPRSEDTIIALVKNAADNGDTIRVIGA-LHSWSDC-AMSD-------GIVVSLDDMQQVLEVDR---------
MBS1617249.1	-MAIISQRAYR----------------------FRNW--AGNQQCT-PSHYYQPETEAELIEVI----KTHSKVRMVGT-GHSWSAL-CHTD-------EALINLDKYNRVITLDR---------
HIA12326.1	-----MNAPHK----------------------FVNW--AATESSN-PEKFLKPSNQEELVEIVNSAINANSKIRVVGA-GHSWSSV-ACTD-------HWLITLDNYCNVLSIDK---------
NEO81287.1	------MNQNK----------------------FINW--SQQYQCI-PTSVHYPASTEEARDIVCQVMQQGTKIRVFGS-GHSPSDI-AMSN-------QDLVCSDRLNQILNINY---------
BAY85903.1	------MNQEH----------------------FINW--SGEYRCT-PSKVHHPRTDVEVREIVCQAIKRGSKIRVFGS-GHSPSDI-AMSD-------EELVCLDGLNTIKDVDY---------
MCP8715902.1	MSSGIPESLKPFLTTKLV---------------HSTW--AGTFLCK-PQAIFQPRNEKEIKELINQARINGKTIMTVGS-GHSPSDL-TMTK-------EWLCNLDKFNQVLKEEPYYGPTSTTD
PSP48796.1	--MLRLTDGV-----------------------WENW--SGSVRAR-PTHVHYPETEATLRDVVERA---DDTLRVAGA-GHSFPPV-ARSA-------ETFVSLQRYTGLDDVDA---------
BAO94255.2	RSLP----PFEPNME------------------VSNW--SSTHSVV-AGKYYQPETVEELKDIVTICHATGQKIRPAGA-MLSPNGI-SLST-------DAMVSLSRLDKVLEVDP---------
XP_844937.1	GQETMSDGT------------------------WTNF--ANIGKCF-PRKHHYPNTVEEVSSIIKVINSAGERCRVVGG-GKSPNSC-TFTN-------GHLIHMDRLNRITSIDE---------
XP_001682394.1	--MSAHSAARR----------------------WTNL--AGIGSCH-PRHHHYPTSTKEVQDAVELVRSQNGKCRVAGA-GKSPNTA-TFTN-------EHLIHMERMNRILSIDT---------
NP_848862.1	--MVHGYKGVQ----------------------FQNW--AKTYGCS-PEMYYQPTSVGEVREVLALARQQNKKVKVVGG-GHSPSDI-ACTD-------GFMIHMGKMNRVLQVDK---------
EDL85374.1	--MVHGYKGVQ----------------------FQNW--AKTYGCS-PEVYYQPTSVEEVREVLALAREQKKKVKVVGG-GHSPSDI-ACTD-------GFMIHMGKMNRVLQVDK---------
OCT81467.1	--MVLGRGGYK----------------------FENW--AQTYGSS-PELYFQPTCVEEIKEILDLARQRSKRVKVVGG-GHSPSDI-ACTD-------DFMIRMDKMNRILKVDK---------
XP_002122023.1	FDISQGKSGYR----------------------FYNW--SKTYSAS-PELYFEPRSDNELRNILSRAKENKKTVKIVGG-GLSPSDI-ACTN-------DFMISLKHLNRVLDVDA---------
XP_005443605.1	ITEVHGQGGVK----------------------FQNW--AKTYGSS-PELYFQPTSVEEIREILDMARQRDKRVKVVGG-GHSPSDI-ACTD-------DFMIQMGKMNRVLKVDK---------
XP_024182616.1	GAATYYSFPFPENAKHKKAQIFRYAPLPEELHTVSNW--SGTHEVQ-TRVFHQPETLEELEKVVKEANENKYRIRPVGS-GLSPNGI-GLSR-------AGMVNLALMDQVLEVDK---------
XP_024165881.1	VTAMLPQSPIQCD-GTGGCNLFNS---------YGVW--NDRKNCH-VPNVTYPRTENELLRAVAYANQNKLKLRVVSQFSHTIPKL-ACPATSTGN--SLLISTSSYDSGIEIDA---------
XP_008240731.1	AAATYYSFPFPENAKHKKAQIFRYAPLPEELHTVSNW--SGTHEVQ-TRVFHQPETLEELEKVVKDAHEKKTRIRPVGS-GLSPNGI-GLSR-------AGMVNLALMDKVLEVDK---------
XP_008220057.1	IQAMPPELPVRCN--KNGCNLYNS---------YGVW--GDRKDCH-VQNVTYPTTEEELRLAVAYANQNKLKVKVVSQFSHTIPKL-ACPATSSGN--SMLISTAKYNSSIEIDA---------
XP_004291192.1	VAAMLPQSPVQCD-GTGGCNLFNS---------YGIW--NDRKDCY-VLNVTYPTTENELLKAVAYANQNKFKLKVVSQFSHTITKL-ACPATTTGN--SLLISTSSYNSGIEIDA---------
XP_004303609.1	GAATYYSFPFPEDAKHKKAQIFRYAPLPEELHTVSNW--SGTHEVQ-TRVFHQPETLEELEKVVKEANARKYRIRPVGS-GLSPNGI-GLSR-------AGMVNLALMDEVLEVDK---------
NP_190376.1	GVATYFSFPFPENAKHKKAQIFRYAPLPEDLHTVSNW--SGTHEVQ-TRNFNQPENLADLEALVKESHEKKLRIRPVGS-GLSPNGI-GLSR-------SGMVNLALMDKVLEVDK---------
NP_182199.1	TISTPPEDPVKCVSGNTNCTVTNS---------YGAF--PDRSTCR-AANVAYPTTEAELISVVAAATKAGRKMRVTTRYSHSITKL-ACTDGTD----GLLISTKFLNHTVRTDA---------
NP_564393.1	ASSTPPDDPVKCESGNNMCTVTNS---------YGAF--PDRSICE-AAKVEYPKTEAELVSIVAAATRAGQKVRVVTRYVHSIPKL-VCTDGKD----GVLISTKFLNNVVGTNP---------
NP_182198.2	AVSTPPDDPVKCVSGNTNCTVTNS---------YGAF--PDRSTCR-AANVAYPKTEAELVSVVAAATQAGRKMRVTTRYSHSITKL-VCTDGTE----GLFISTKFLNHTVQADA---------
NP_182197.1	VISTPPEDPVKCVSGNTNCIVTNS---------LGAF--PDRSTCR-AANVAYPTTEAELVSIVAAATKAGRKMRVTTRYSHSIPKL-TCTDGND----GLFISTKFLNHTVQADA---------
NP_200460.1	ASSSPPDDPVNCEFGNTMCTVTNS---------YGAF--PDRSICK-AAKVEYPRTEAELVSIVAAATRAGQKMRVVTRYSHSFPKL-VCTDGKD----GTLISTKFLNHVVTTNP---------
XP_023896433.1	VRCSPPEDHIKCSSKNTDCTITNS---------YGVF--PDRSTCR-AAEVAYPSTEEELISIVAKATENKRKMKVATRYSHSIPKL-VCPDGED----GLLISTKYLNHALKIDV---------
XP_023896434.1	VCCIPPEDPIKCSSDNTDCTITNS---------YGAF--PDRSTCR-ATEVAFPSTEQELISIVSKASRNKRKMKVATRYSHSIPKL-VCLDGQD----GLLISTKYLNRTLKVDA---------
XP_023900169.1	GVATYYSFPFPENAKHKKAQFFRYAPLPDDLHTVSNW--SGTHEVQ-TRVFHQPENLQQLENVVKEAHKAKTRIRPVGS-GLSPNGI-GLSR-------AGMVNLALMDKVLEVDK---------
XP_023914113.1	IYAMPPQSPVQCS-TTGGCTLYNS---------YGVW--GDRKDCH-APNVTYPTTEQELRSVVAYANQNKLKVKVVTKFSHTIPKL-ACPTSNSEA--SLLVSTEKYNSNIVVDK---------
XP_043131130.1	LLHAVFSSAYR----------------------WFNW--QFEVTCE-SDAYIAPEDESAAASFLKDQYARGSLIKVVGN-GHGFGNLTTCVDTARTKKPSYIVSLTNLKH-LDINR---------
XP_002422445.1	-MADIPESLKPFVTNKLI---------------HSTW--AGTFLCK-PQAIFQPRNVEEIQELIKQARLHKKTIMTVGS-GHSPSDL-TMTK-------EWLCNLDKFNHVLLEQPYYAPKSITD
NP_013624.1	MSTIPFRKNYV----------------------FKNW--AGIYSAK-PERYFQPSSIDEVVELVKSARLAEKSLVTVGS-GHSPSNM-CVTD-------EWLVNLDRLDKVQKFVEY--------
XP_003651626.1	ASPIPPELPPE----------------------LASL--PAE-----------PESVAEVEHAVTLARQCRRRLVTVGS-GHSPSDL-TCTS-------SWMLNLDRLAGVLTADP---------
XP_049145240.1	---MPSLRPLPLLAMLLASTAVTAAP-------YNTFDGPGFPACNDVAAVHNATSVSDIQSLVKDAIASGQKVRASGK-GHMWYDT-MCSDDAN----TVIIRTEDVNGISEFDL---------
NP_055577.1	LFLLPLSLIFDIYYY------------------VRAW--VVFKLSS-APRLHEQR-VRDIQKQVREWKEQGSKTFMCTG-RPGWLTVSLRVGKYKKTHKNIMIN---LMDILEVDT---------
XP_004926865.1	LALLPMSAAWKLWSI------------------IRNY--VVFKMNS-APKMHDDK-VKEVQRQIKEWLSGDKSTHLCTA-RPTWQTMSFRHSMYKRTFTNIQIN---LVDVLEVDK---------

Oxygen reactivity marker (G) region

```
                 216 220          230            240          250          260          270           280          290          300         310          320          330          340
NP_216287.1      -----PTGLVTVEGGAKLRALGPQ-LAQRRLGLENQGDVDPQSITGA-TATATHGTGVRF-Q-NLSARIVSLRLVTAGG------EVLSLSEGDD---YLAARVSLGALGVISQVTLQTVPLFTL
MBI0379321.1     -----EAGTVTVAAGTSLRQLNET-LSAHSLSLVNMGDIMEQTVAGA-TSTGTHGTGRDS-A-SIAAQIKGLELVTADG------SVLRCSAEENPEIFAAARIGLGALGVVSEITFGVEPEFLL
NP_254014.1      -----VTLQAEFGGGTPMSQMGAP-LKEIGQALVNMADIDYQTLAGA-IATSTHGTGVGF-G-SYSAQVRGLQLVTASG------EVLECDAKRNVEVFDAARVSLGALGVVTRVRLQNRAAYRL
MCO4763851.1     -----DGLRVRVQAGIRLARLNEE-LAAVGLALPIVGSVVEQSIAGV-MSTGTHGSSLTH-G-NIPSAVVGMRLVTGKG------DVLVL--DEDHELLPAARVALGALGVITEVTLSVQPAFRL
MBS1617249.1     -----EQKLLTVQAGIKLWQLNDY-LDTEGLALINLGSIAQQSIAGA-ISTATHGSGIQY-R-ILASQVQRFTLIQADG------TPLTLDRDADKETFDLAIISLGALGVVSEITIHICEAFRL
HIA12326.1       -----EAKQITVEAGLRIKELNVI-LKDNGLALSNLGSISEQSIAGA-ISTGTHGTGIKF-S-ILSTQILALTLVNGKG------ELVSLDHEKDADLFKAALVSLGALGIISTVTIQCEELFNL
NEO81287.1       -----DSQTVVTQAGVTLKDLNQS-LAKHRLALPNLGSISAQTVGGA-MATATHGTGLNY-G-VLSTIIQEITLVTGLG------EVIKISKDENSQLFNAAKCHLGSLGLVTELKLQVTKEFDL
BAY85903.1       -----ENQTVIVEAGVTIYELSQN-LARYGLALPNLGSISAQTISGA-MATGTHGTGLGY-G-TMPTIIEEITLVNGKG------EIIKVSENENSQFFNAVKCHLGSLGLITEYKLKVTESFDL
MCP8715902.1     DTPEVKFVDLTVEAGTRVFELNEY-LKKHDLAIQNLGSISDQSVAGL-ISTGTHGSTQYHG--LVSQQVVSITFLNSAG------ESITCSSIEKPEYFKAILLSLGKIGIITHVTLRTCPKYTI
PSP48796.1       -----ETGRVTVRAGTPLSALTDA-LADHGLMLENMGDIDAQTVAGA-LSTGTHGTGTDF-G-VMPTQAAAMRLVTADG------DVLNL--ETDDDRFPLAQVSLGTLGVVSTVTLDVVPDYRL
BAO94255.2       -----KGQTITVQAGIKVSQVLEE-LRKHGLTLQNFSSIQDQQMGGW-TQVAAHGTGATLP--TVEEMIVRMKLVTPSL------GELELSEQHEPELFQMAKVGLGCLGIVSEMTLRCIPQHKL
XP_844937.1      -----KSMTIVCEGGALISDVFER-LSAHDLMLRCVPSFVQTTVGGV-IATATHSSGIRSR--SISDCVVRLQLVDGRG-------ILHTFDASTPKELSLSACHLGMLGVVVSVTLQAEKKRLW
XP_001682394.1   -----VAHTITCEAGAVMEEVMRS-VDKVGLMVRCVPSYVRTTVGGC-IATATHSSGIQCH--CLSDYVRGLKIVDGCA------QIRTLVAGKDDAELRLAACHLGVMGIVTEVTLEVQPRIQW
NP_848862.1      -----EKKQVTVEAGILLTDLHPQ-LDKHGLALSNLGAVSDVTVGGV-IGSGTHNTGIKH-G-ILATQVVALTLMKADG------TVLECSESSNADVFQAARVHLGCLGVILTVTLQCVPQFHL
EDL85374.1       -----EKKQVTVEAGILLADLHPQ-LDEHGLAMSNLGAVSDVTVAGV-IGSGTHNTGIKH-G-ILATQVVALTLMTADG------EVLECSESRNADVFQAARVHLGCLGIILTVTLQCVPQFHL
OCT81467.1       -----EKKQVTVEGGMLLTDLNKE-LDKRGLALSNLGAVSEVAAAGV-IGTGTHNTGITH-G-ILSTQVVTLTLMTALG------EILECSEASNPEIFQAARLHLGSLGVILSVTIQCRSAFHL
XP_002122023.1   -----KRCTVTVEAGVTINELNENILPSHGLSLINLGSVSGQTVGGI-IGTGTHGSGEKF-G-SFATHVLELVLMKADG------TILRCSQQENAEIFSAACCHLGILGIILSVKLQCEAAFML
XP_005443605.1   -----EKQQVTVEGGIFLSDLNVE-LSKHGLALANLGAVSEVAAAGV-IGTGTHNTGIKH-G-ILSTQVVALTLLTASG------EILECSESINADIFQAARLHLGCLGIVLTVTFQCVPQFHL
XP_024182616.1   -----EKKRVRVQAGIRVQQLVDG-IKDQGLTLQNFASIREQQIGGI-VQVGAHGTGARLP--PIDEQVISMKLVTPAK------GTIEVSKEKDPELFYLARCGLGGLGVVAEVTIQCVDRQEL
XP_024165881.1   -----ANLAVTVDGGVGLRKLIDT-VEAAGLSLVAAPYWEGVSIGGL-ISTGSHGSSWWGKGGSVHDHIVGLSLIVPAKQSEGYAKILRL--EPKDQLFNAAKVSMGLLGVISKVKLLLEKGFKR
XP_008240731.1   -----EKKRVRVQAGIRVQELVDG-IKEHGLTLQNFASIREQQIGGI-LQVGAHGTGAKLP--PMDEQVISMKLVTPAK------GTIEISKEKDPELFYLARCGLGGLGVVAEVTIQCVERQEL
XP_008220057.1   -----KNLAVTVDAGIGLRRLIDA-VEEAGLSLVAAPYWEGVSVGGL-VSTGAHGSSWWGKGGAVHDHIVGLSLIVPAKQSEGFAKIVKL--EPKDQIFNAAKVSLGLLGVISKVKLSLEPRFKR
XP_004291192.1   -----ANLAVTVDSGVGLRKLIDT-VEAAGLSLVAAPYWEGVSIGGL-ISTGSHGSSWWGKGGSVHDHIVGLSLIVPAKQPEGYAKILRL--EPKDQLFNAAKVSLGLLGVISKVKLSLEKGFKR
XP_004303609.1   -----EKKRVRVQAGIRVQQLVDG-IKDQGLTLQNFASIREQQIGGI-LQVGAHGTGARLP--PIDEQVISMKLVTPAK------GTIEVSKEKDPELFYLARCGLGGLGVVAEVTLQCVERQEL
NP_190376.1      -----EKKRVTVQAGIRVQQLVDA-IKDYGLTLQNFASIREQQIGGI-IQVGAHGTGARLP--PIDEQVISMKLVTPAK------GTIELSREKDPELFHLARCGLGGLGVVAEVTLQCVARHEL
NP_182199.1      -----TAMTLTVESGVTLRQLIAE-AAKVGLALPYAPYWWGLTVGGM-MGTGAHGSSLWGKGSAVHDYVTEIRIVSPGSVNDGFAKVRVLRETTTPKEFNAAKVSLGVLGVISQVTLKLQPMFKR
NP_564393.1      -----EAKTLTVESGVTLRQLIGE-AAELELALPHAPYWWGLTVGGL-MGTGAHGSSLWGKGSAVHDYVSEIRMVSPGLASDGYVKVRVLSETIDPDEFRAAKVSLGVLGVISQVTFQLQPMFKR
NP_182198.2      -----TAMTMTVESGMTLRQLIVE-AAKVGLALPYAPYWWGLTVGGM-MGTGAHGSSLWGKGSAVHDYVTEIRMVSPGSVNEGFAKIRILSETTTPNEFNAAKVSLGVLGVISQVTFELQPMFKR
NP_182197.1      -----KAMTLTVESGVTLRQLIAE-AAKVGLALPYAPYWWGVTVGGM-MGTGAHGSSLWGKGSAVHDYVTEIRMVSPGSVNDGFAKIRVLSETTTPNEFNAAKVSLGVLGVISQVTFELQPMFKR
NP_200460.1      -----EAKTLTVESGVTLRQLIEE-AAKFDLALPYAPYWWGLTVGGM-MGTGAHGSSLWGKGSAVHDYVTEIRLVSPGLASDGYVKVQVLSETIDPEEFRAAKVSLGVLGVISQVTFELQPMFKR
XP_023896433.1   -----EAMTITVESGVTLRQLINE-AAKAELVLPYAPYWWGLTIGGL-LSTGAHGSTLRDKGSAVHDYLTELRIVSPGGAEDGYAKVREL--KDGDQDFNAAKVSLGVLGVISQVTLKLQPMFKR
XP_023896434.1   -----EAMTITVESGVTLRQLINE-AAKAGLALPYTPYWWGLTIGGL-LSTGAHGSTLWANGSSVHDYLLEVRIVTTGFA--GFARVRSL--KDGDKDFNAAKVSLGVLGVISQVTLELQPMFKR
XP_023900169.1   -----GKKTVRVEAGIRVQQLVDG-IKDSGLTLQNFASIREQQIGGI-VQVGAHGTGARLP--PIDEQVISMKLVTPAK------GTIEVSKEKDPELFYLARCGLGALGVVAEVTIQCVERQEL
XP_023914113.1   -----DNLVVTVDAGVGLRELIDK-VEEAGLSLVAAPYWEGVSVGGL-ISTGAHGSSWWGKGGAVHDYVIGLSLIVPARPSEGYAKIIRL--EALDPLLNAAKVSLGLLGVISKVKLSLERGFKR
XP_043131130.1   -----EDMTVTFGAGWDVYDLIQE-LKANGLSFNNLGVEQVQNFIGA-VSTGTHGTGSHV-G-NMATQVVGLRVLDSQG------SLRVIDQTTNAEELKAFRVSLGALGLITEMTIKVHPTYLV
XP_002422445.1   KTPEIKFVDLTVESGIRIFELNEY-LKRNNLAIQNLGSISDQSIAGL-ISTGTHGSTQYHG--LISQQVVSVKFLNSIG------ELITCSSIDKPEYFRAILLSLGKIGIITHITLRTCPKYTI
NP_013624.1      --PELHYADVTVDAGMRLYQLNEF-LGAKGYSIQNLGSISEQSVAGI-ISTGSHGSSPYHG--LISSQYVNLTIVNGKG------ELKFLDAENDPEVFKAALLSVGKIGIIVSATIRVVPGFNI
XP_003651626.1   -----ATGLCTVQAGIRLRALSAA-LAAAGLALPSLGSIDEQSIAGA-ISTGTHGSSLRHG--LVSECVVALKVTLADG------KTRECSPQQRPELFRAALLSVGALGVVVEVTLRAVPAFSL
XP_049145240.1   -----EGGSVVIEAGVTFLQLAEY-LHARGASAGYTLVNWNITLAGC-VAMGAHRSSIREDS-MVAAGVLELDIIDGNG------ELRHLVRDNNDDTWLAASTSLGLLGVIVRMKFKIYPDFKV
NP_055577.1      -----KKQIVRVEPLVTMGQVTAL-LTSIGWTLPVLPELDDLTVGGLIMGTGIESSSHKY-G-LFQHICTAYELVLADG------SFVRCTPSENSDLFYAVPWSCGTLGFLVAAEIRIIPAKKY
XP_004926865.1   -----ENMTVRCEPLVTMGQLSRT-LAPLGLALPVVPELDQLTVGGLVMGTGVETTSHVH-G-LFQHVCLEYELVLADG------SVVNCSKDENADLFYAVPWSYGTLGFLTSVVIKVIPAKKY
```

```
                 341   350         360         370         380         390         400         410         420         430         440         450         460 465
NP_216287.1      HRHDQ----RRSLAQTLER--LD-EFVDGNDHFEFFVFPYADKALTR----------------------TMHR--SDEQP---KP----------------------------------------
MBI0379321.1     SAREE----PMPFDRVMAD--FE-QLVAENEHFEFYWFPHTGNCNTK----------------------RNNR--STGPA---APVG--------------------------------------
NP_254014.1      RERQW----IARTEELLED--VE-KNTRENQHWEMLVVTHSDYALSI----------------------ALNE--TDEPK---TPPL--------------------------------------
MCO4763851.1     CETTE----TIPFDDAVRD--ME-AIARSAEYVKLWWLPHTDAVIVF----------------------RSER--TTEPG------R--------------------------------------
MBS1617249.1     HDRTY----SMDFDKMLEK--LD-DLVTTTDHFKMWWFPHVKDIVLY----------------------QYQR--TTAPA---N-----------------------------------------
HIA12326.1       EEFAY----PVEFDEGVKR--IP-ELLKEHEHMKLWWFPHVKELQVY----------------------CQNRTKAEHLN---------------------------------------------
NEO81287.1       EVREQ----PETLETVLDK--LP-ERLQ-ADHYRFWYLPHADRVWEW----------------------SATRKPPGKTA------P----------------------------------KQNF
BAY85903.1       EVKEQ----PQSLATVFEN--LP-ESLK-SDHYRFWYLPHVDMAWEW----------------------TATRQLPEEST------V----------------------------------KKNI
MCP8715902.1     KSKQE----IINFDTLLSN--WD-NIWLELEFIRIWWFPYTNKCVLW-------------------------R--ANKST---DPLS--------------------------------------
PSP48796.1       RERTF----PADVEAVLAD--LE-AY-RECRNFEFWWFPHTDTALVK----------------------TLDE--TDAEG---APGR----------------------------------IDGL
BAO94255.2       LETTY----VTDIAGIKKD--HE-ELLRKYRHVRYMWLPRTGKVVVV----------------------VSNP---HAGG---QPLL----------------------------------QKVS
XP_844937.1      RIESR----PIPFRKLTEGDTLK-KRIAESEFYRFFWMPNTDQCYESTAEFVGEEGADQTKRVDESIKLAMGK--KHEAT---LPMTAGNTITKLTSSKLRNFSSEKCNSTGEDYQMWLRNQRTL
XP_001682394.1   KLVSQ----PLLMKDATNAALVA-EKVKSTEYYRWWWVPHTDGCYES----------------------YGRV--ESTTGISALPPL------------PDALTAQDDAPVQLSPAALQEESRTN
NP_848862.1      LETSF----PSTLKEVLDN--LD-SHLKKSEYFRFLWFPHSENVSII----------------------YQDH--TNKEP---------------------------------------------
EDL85374.1       QETSF----PSTLKEVLDN--LD-SHLKRSEYFRFLWFPHTENVSII----------------------YQDH--TNKAP---------------------------------------------
OCT81467.1       KEVPF----SSTLQEVLDN--LD-THLNSSEYFRFYWFPHTENVSVF----------------------YQDP--TDKPP---------------------------------------------
XP_002122023.1   HEKKA----SSKLETVLID--LN-EHVSSAQHFQFVWFPHTDNVVTI----------------------ARNR--TRRLK---------------------------------------------
XP_005443605.1   HEVAF----PSTLTEVLDH--LE-DHLKRSQYFRFLWFPHSENVSII----------------------YQDP--TNKPP---------------------------------------------
XP_024182616.1   VEHTT----VSNMEEIKKN--HK-KLLSENKHVKYLYIPYTDTVVVV----------------------TCNP--VSKWK---GPPK-------------------------------FKPKYTT
XP_024165881.1   SVSYD----FTDDDQIENK--YM-DHGKKYEFADITWYPSKHTAAYR----------------------YDNRVPLNISG---DGVY-------------------DFLGFQANPILVSKSVRAS
XP_008240731.1   VEHTS----VSNMEEIKKN--HK-KLLSENKHVKYLYIPYSDTVVVV----------------------TCNP--VSKWK---GPPK-------------------------------FKPKYGS
XP_008220057.1   SIAYD----FTDDAQIENI--YM-DHAKKYEFADITWYPSKHTAAYR----------------------YDNRVPLNASG---DGTY-------------------DFLGFQSNSILISKSIRAT
XP_004291192.1   SITYD----FTDDDQIEDK--YM-VHGKKYEFADITWYPSKHTAAYR----------------------SDNRVPLNASG---DGVY-------------------DFLGFQANPILVSKSVRAS
XP_004303609.1   VEHTT----VSNMEEIKKN--HK-KLLSENKHVKYLYIPYTDTVVIV----------------------TCNP--VSKWK---GPPK-------------------------------FKPKFTT
NP_190376.1      VEHTY----VSNLQEIKKN--HK-KLLSANKHVKYLYIPYTDTVVVV----------------------TCNP--VSKWS---GPPK-------------------------------DKPKYTT
NP_182199.1      SLRYV----MRNDSDFGDQ--AV-TFGMKHEFADFIWLPSQGKVVYR----------------------MDDRVAVNTSG---NGLL-------------------DFMPFRSQLSAALAIIRSS
NP_564393.1      SLTFV----MQNDSDFGDQ--AV-TFGEKHEFADFLWLPSQGKVVYR----------------------MDDRVPVNTSG---NGLF-------------------DFFPFRPQLSVALAIIRSL
NP_182198.2      SLTYT----MRNDSDFEDQ--AV-TFGKKHEFADFIWLPSQGKVVYR----------------------RDDRVAVNTSG---NGLF-------------------DFLPFRSQLSAAIAIIRTS
NP_182197.1      SLKYV----MRNDLDFNDE--AL-TFGKKHEFADFVWLPSQGKVVYR----------------------MDDRVAVNTLG---NGLY-------------------DFFPFRSQLSAVLATTRSS
NP_200460.1      SLNYV----MRNDSDFGDQ--AV-SFGERHEFADFLWLPSQGKVVYR----------------------MDGRVPLNTSG---DGLF-------------------EFFPFRSQLSLVLAIDRSL
XP_023896433.1   SITYL----KKNDSDLGDM--VA-SFGNEHEFADITWYPSQRKAVYR----------------------IDDRVYTNTHGHGNNGLY-------------------DFTPFRATASVELGIVRTT
XP_023896434.1   SITYL----TKDDSDLGDK--VT-SFGNEHEFADITWYPSQRKAVYR----------------------IDDRVNTNSPG---NGLY-------------------DFTPFRSTASIALGAIRTT
XP_023900169.1   VEHTT----VSNVKEIKKN--HK-KLLSENKHVKYLHIPYTDTVVVV----------------------TCNP--VSKWR---GPPK-------------------------------FTPKYSS
XP_023914113.1   SVTYN----FTNDDHFEDV--FM-NHAKKYEFADITWYPSKNTAVYR----------------------YDERVPLNVSG---NGVY-------------------DFIGFQSNLILISKSIRAT
XP_043131130.1   KKTTRVLNATTDYAQMYKE--IA-QLYKEHDRMTV-WGPHFD---------------------------WDSK--AQDWA--IEPTY--------------------------------------
XP_002422445.1   KSKQE----IINFETLLNN--WN-NIWLESEFIRIWWFPYTNKCVLW-------------------------R--ANKSI---DPLS--------------------------------------
NP_013624.1      KSTQE----VITFENLLKQ--WD-TLWTSSEFIRVWWYPYTRKCVLW----------------------RGNK--TTDAQ---NG----------------------------------------
XP_003651626.1   AWSQT----IDADARLFST--WEGELWKQAEFVRVWWFPYMRRAVVW----------------------HADKVPREDIE---SGRV--------------------------------------
XP_049145240.1   YAQQK----TLEESEVLDG--DIYGMIAPYATANFWWWPYKRKFHHR----------------------YYDVVPTNTSD---------------------------------------------
NP_055577.1      VKLRF--EPVRGLEAICAKFTHE-SQRQENHFVEGLLYSLDEAVIM-----------------------TGVM--TDE-A---EPSK----------------------------------LNSI
XP_004926865.1   VRIHY--YPFNNLSDLAKRFSQESLKPNPHQFVEALLYTRDSGVLM-----------------------LGDM--VDEVG---RDGK----------------------------------LNPI
```

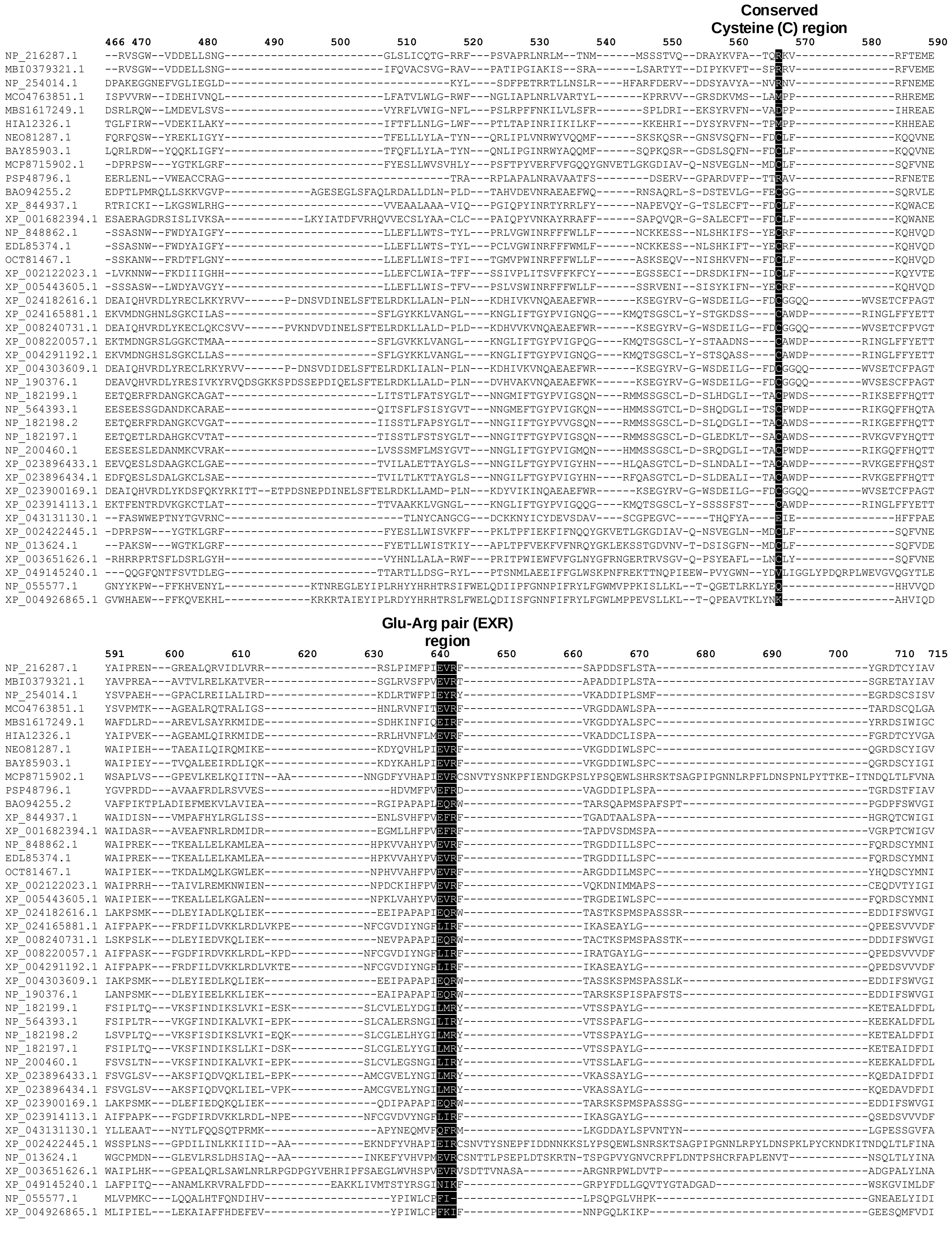

Conserved
Cysteine (C) region
466 470 480 490 500 510 520 530 540 550 560 570 580 590
NP_216287.1
MBI0379321.1
NP_254014.1
MCO4763851.1
MBS1617249.1
HIA12326.1
NEO81287.1
BAY85903.1
MCP8715902.1
PSP48796.1
BAO94255.2
XP_844937.1
XP_001682394.1
NP_848862.1
EDL85374.1
OCT81467.1
XP_002122023.1
XP_005443605.1
XP_024182616.1
XP_024165881.1
XP_008240731.1
XP_008220057.1
XP_004291192.1
XP_004303609.1
NP_190376.1
NP_182199.1
NP_564393.1
NP_182198.2
NP_182197.1
NP_200460.1
XP_023896433.1
XP_023896434.1
XP_023900169.1
XP_023914113.1
XP_043131130.1
XP_002422445.1
NP_013624.1
XP_003651626.1
XP_049145240.1
NP_055577.1
XP_004926865.1
Glu-Arg pair (EXR)
region
591 600 610 620 630 640 650 660 670 680 690 700 710 715

```
                                       HWXK motif
                                         region
               716 720       730       740       750       760       770       780       790       800       810       820       830       840
NP_216287.1    HQY--------AG-MEFESYFRA---VEEIMD-DYAG----RPHWGKRHY---------QTAAT----------------------------LRERY-PQWDRFAAVRDRLDPDRVFL-------
MBI0379321.1   HLY--------RG-TPHRAYFTA---AEQIMI-AHGG----RPHWGKLHS---------RDAAY----------------------------LAGVY-PRFGEFAAVRDRLDPDRLFT-------
NP_254014.1    HQH--------YS-MDHHNFFAA---VEPIFW-KYAG----RPHWGKLHG---------LNAHQ----------------------------LQGLY-PRWKAFAEVRQALDPRGRFL-------
MCO4763851.1   YMA--------ES-EGIERYFGA---FQGAMK-ALGG----RPHWGKEFQ---------ANVAE----------------------------LSGQY-PEFERFVALARRLDPKQIFR-------
MBS1617249.1   YLI--------GD-RGWDALLHD---FEALAV-KYNG----RPHWGKEYH---------IDRAY----------------------------LRSQY-PQVDAFCALRHRLDPDHKFS-------
HIA12326.1     YKA--------GD-RGWKEYLHG---FEEIMA-AYDG----RPHWGKEFS---------IDKNA----------------------------IGQLY-PELERFQAVREQMDPNNVFA-------
NEO81287.1     ITY-MPY---GKY-VDCQSYFDD---YEKIMA-KLDG----RPHWAKKFG---------PDADK----------------------------LEKMY-PHWNDFQKVRSQLDPDNIFG-------
BAY85903.1     INY-MPY---GKR-VDCRDYFND---YEKIMA-KFDG----RPHWAKRFG---------PDADK----------------------------LAKIY-PHWNDFQQVRYQLDPDNLFG-------
MCP8715902.1   TMY-RPF---GTN-VETHQWFQL---FENVMS-KAGG----KPHWAKNFIG--------LTQDEKYNEKEDLKTQLEFGGKPFYSMIGFKPVMKDWFGEDLVKFNNVRKETDPHGVF--------
PSP48796.1     HAY--------HR-REWETLVRE---AEAVFD-RYDG----RPHWGKHHT---------KTAAE----------------------------FAGLY-PEFETFREARRDLDPEGLFL-------
BAO94255.2     IMYNTPGQDEQGLEKVAKAFKSYCAALEGVHQ-EYGA----VPHWAKVEP---------CDDPA--------------------AAQRLRDRLEARY--PIAKFKYMRAQLDPKNILS-------
XP_844937.1    VMY-RPY---LRHARDTLRYYDA---FSKAMT-VMGG----RPHWAKYYT---------WGPEQ----------------------------MLKAYGKNWEDFLLFRKKMDPEGVFL-------
XP_001682394.1 VMY-RPY---GQEARDTRRCYDG---FCRVME-EMGG----RPHWAKYYD---------WGHRE----------------------------ITATYGDHWERFLALRRRMDPDDIFV-------
NP_848862.1    IMY-RPY---GKD-VPRLDYWLA---YETIMK-KFGG----RPHWAKAHN---------CTRKD----------------------------FEKMY-PAFHKFCDIREKLDPTGMFL-------
EDL85374.1     IMY-RPY---GKD-VPRLDYWLA---YETIMK-KFGG----RPHWAKAHN---------CTRKD----------------------------FEEMY-PTFHKFCDIREKLDPTGMFL-------
OCT81467.1     IMY-RPY---GKD-VAHQEYWVE---YENIMK-KVGG----RPHWAKAHT---------CTRKD----------------------------FEKMY-PAFSTFKSIREKLDPTGMFL-------
XP_002122023.1 ISY-RPY---GKL-VPHDKWFTF---YENLAL-KYGG----RPHWAKDHK---------VNSQT----------------------------FQQIY-PNFNKFMKLRSELDPDNLFL-------
XP_005443605.1 IMY-RPY---GKN-VPRLNYWLT---YEGIMK-KYGG----RPHWAKAHS---------CTRKD----------------------------FEKMY-PAFPKFCSIREKLDPTGIFL-------
XP_024182616.1 IMY-LPTTDARQRKDITEEFFHYRHLTQTRLWDTYGG----RPHWAKIEV---------PKDKE--------------------QLTALQARLRKRF--PVDAYNKARTELDPNRILS-------
XP_024165881.1 NYY-RAD-DASTP-RFDQDVWEE---VEQLAFFKHSS----YEHWAKNRN---------MAFLN----------------------------VQSKY-SKFTKFVGVKRQLDPQNMFS-------
XP_008240731.1 IMY-LPTTDARQRKEITEEFFHYRRLTQVQLWDKYGA----KPHWAKIEV---------PKDKE--------------------ELASLQARLRKRF--PVDAYNKARKELDPSRILS-------
XP_008220057.1 NYF-RAD-DALTP-RFNQDVWEE---VEQLAFFKYSA----YEHWAKNRN---------LAFLD----------------------------VQKKY-PNFNKFVAVKRQLDPQNLFS-------
XP_004291192.1 NYY-RAD-DALTP-RFDQDVWEE---VEQLAFFKHGA----RPHWAKNRN---------IAFLN----------------------------VQNKY-SKFNKFVGVKRQLDPQNMFS-------
XP_004303609.1 IMY-LPTTDARQRKDITEEFFHYRHLTQTRLWDTYGA----KPHWAKIEV---------PKDKE--------------------QLTTLQARLRKRF--PVDAYNKARSELDPNQILS-------
NP_190376.1    IMY-LPTADPRQRKDITDEFFHYRHLTQKQLWDQFSS----YEHWAKIEI---------PKDKE--------------------ELEALQARIRKRF--PVDAYNKARRELDPNRILS-------
NP_182199.1    TYY-RAK-DPLSP-RLYEDFIEE---IEQIALFKYSA----YEHWGKNRN---------LAFDG----------------------------VIKKY-KNVPAFLKVKESYDPMGLFS-------
NP_564393.1    TYY-RSKDDPLAP-RLYEDFIEE---IEQMAIFKYNA----LPHWGKNRN---------LAFDG----------------------------VIRKY-KNANTFLKVKERFDPLGLFS-------
NP_182198.2    TYY-RAK-DPLTP-RLYEDFIEE---IEQIALFKYNA----LPHWGKNRN---------LAFDG----------------------------VIRKY-NNAPAFLKVKDSYDPKGLFS-------
NP_182197.1    TYY-RAN-DPLTP-RLYEDFIEE---IEQIALLKYNA----LPHWGKNRN---------LAFDG----------------------------VIKKY-KNAPAFLKVKESYDPNGLFS-------
NP_200460.1    TYY-RSKNDPLAP-RLYEDYIEE---IEQMAIFKYNA----LPHWGKNRN---------LAFDG----------------------------AIRKY-KNANAFLKVKEKFDSLGLFS-------
XP_023896433.1 TYY-RSK-DPLTP-RLFEDILEE---VEQLALFKYNA----LPHWGKNRN---------IAFDG----------------------------VINKY-KNAKEFLKVKKVYDPFGLFS-------
XP_023896434.1 TYY-RSK-DPLTP-RLYEDILEE---VEQLALFKYGA----LPHWGKNRN---------VAFDG----------------------------VINKY-KNAKEFLRVKKAYDPFGLFS-------
XP_023900169.1 IMY-LPTMDARQRKEITEEFFHYRHLTQSRLWDRYSA----YEHWAKIEV---------PKDKE--------------------ELAALQARLRKRF--PVDAYNKARRELDPNKILS-------
XP_023914113.1 NYY-RAS-EASTP-RLNQDVWEE---VEQMAFFKYGA----KPHWAKNRN---------LAFLD----------------------------VQHKY-PNFNRFIAARKQLDPQNMFS-------
XP_043131130.1 VLE-IDW---MQEYNNFTTLWQNQELAEEFLP-RFGEAYNVRSHWNKMSP---------ANSTY----------------------------ILDKF-PKLPEFLAIQERQDPKCQFV-------
XP_002422445.1 TMY-RPF---GTN-VETHKWFQL---FENVMS-KAGG----KPHWAKNFIG--------LTQDEKYDKLSDLKTQLEFGGKPFYTMLGFKPVMKNWFGKDLIDFNNVRKQTDPEGVF--------
NP_013624.1    TIY-RPF---GCN-TPIHKWFTL---FENTMM-VAGG----KPHWAKNFLGSTTLAAGPVKKDTDYDD---------------FEMRGMALKVEEWYGEDLKKFRKIRKEQDPDNVFLANKQWAI
XP_003651626.1 TMY-RPYFRDPTG-EATERYYAA---FEWLMR-DLGG----RPHWAKTFT---------VSPDE----------------------------FASWYGDDWRAFRQVRDAADPEGMFV-------
XP_049145240.1 PTF-RPT--VGDHSRFNEPFYHN---LAKVLIDEFPC----RPHWTKNTR---------EVFAQ---------------------------AAKNIDPDHIARFKAVREKFDPNGMF--------
NP_055577.1    GAYGEPR---VKH-FEARSCMRQ---LEKFVRSVHGF----QMLYADCYM----------NREE----------------------------FWEMF--DGSLYHKLREKL-GCQDAF-------
XP_004926865.1 GVYGVPK---AKG-FETIASTRH---VESFVIQNQGF----QMLYADTYT----------TREE----------------------------FRQMF--DHKLYDRVRASLPNCKEAF-------


               841      850       860       870       880       890       900       910       920       930
NP_216287.1    -------NDYTRRVLGPZ------------------------------------------------------------------------
MBI0379321.1   -------NDYLRRVLGDZ------------------------------------------------------------------------
NP_254014.1    -------NAHLSSILGVTZ-----------------------------------------------------------------------
MCO4763851.1   -------NRFVARLFESTEEEIHZ------------------------------------------------------------------
MBS1617249.1   -------NTMIDAICGTIZ-----------------------------------------------------------------------
HIA12326.1     -------NRFVREYIMZ-------------------------------------------------------------------------
NEO81287.1     -------NSYSDRVLGKPQKTALSYSZ---------------------------------------------------------------
BAY85903.1     -------NSYSDRVLGKRGRZ---------------------------------------------------------------------
MCP8715902.1   ----------------LSGKVWAERNGILLDZ----------------------------------------------------------
PSP48796.1     -------NEHLRDVFGAZ------------------------------------------------------------------------
BAO94255.2     -------NDLIERVIGHZ------------------------------------------------------------------------
XP_844937.1    -------NGWFNSLSGNSPVLNSTISHLZ-------------------------------------------------------------
XP_001682394.1 -------NGWFRNLMSPDRV-NSTACTPZ-------------------------------------------------------------
NP_848862.1    -------NSYLEKVFY--------------------------------------------------------------------------
EDL85374.1     -------NSYLEKVFY--------------------------------------------------------------------------
OCT81467.1     -------NAYLEKVFY--------------------------------------------------------------------------
XP_002122023.1 -------NEYWKRILK--------------------------------------------------------------------------
XP_005443605.1 -------NSYLEKVFY--------------------------------------------------------------------------
XP_024182616.1 -------NIKLEKLFPLSDTIZ--------------------------------------------------------------------
XP_024165881.1 -------SEWSDEI--VFGKEGAKSDGCGLEGMCICSEDRHCSPGKGYFCKPGVVYEEARVCRYSPSSNVESDSPKTDLZ----------
XP_008240731.1 -------NNKLEKLFPLSDTIZ--------------------------------------------------------------------
XP_008220057.1 -------SEWSDEV--VFGKEGVKGDGCALEGQCICSEDRHCSPGKGYFCKPGLVYQEARVCRYSSSPPSPFSVGSGSDKNDLZ------
XP_004291192.1 -------SEWSDEV--VYGKEGGKSDGCGLEGMCICSEDRHCSPGKGYFCKPGLVYEEARVCRYSTSSNVASDNPKTDZ-----------
XP_004303609.1 -------NVKLEKLFPSSDTIZ--------------------------------------------------------------------
NP_190376.1    -------NNMVEKLFPVSTTAZ--------------------------------------------------------------------
NP_182199.1    -------SEWTDQILGIKGNVTIIKDGCALEGLCVCSEDAHCAPTKGYFCRPGKVYKEARVCTRADD-ISVIQSLSYZ------------
NP_564393.1    -------TEWTNQILGLKGNVTIVKEGCALEGLCVCSDDAHCAPKKGYLCRPGKVYTKARVCTHVKSVNGYDDHTKFNLMFNRIZ-----
NP_182198.2    -------SEWTDQILGIKGNASIVKDGCALEGLCICSKDAHCAPAKGYLCRPGKVYKEARVCTRVDGIISVIZ-----------------
NP_182197.1    -------SEWTDQILGIKGNPTIVKDGCALEGLCICSDDAHCAPSKGYLCRPGKVYKEARVCTHVPKZ----------------------
NP_200460.1    -------TEWTDQILGLKGNVTIVKQGCALEGLCICSEDSHCAPTKGYLCRPGKVYREARVCTRVSSZ----------------------
XP_023896433.1 -------SEWTDQVLGLKNSVTIDKEGCALEGLCICSQDIHCAPKKGYYCSPGKVYKDARVCARVTSKMNMCEEEQWGSQDFTFKGIKIZ
XP_023896434.1 -------SEWTDQVLGLKNSVTIDKEGCALEGLCICSQDIHCAPNKGYYCRPGKVYKDARVCARVTSKZ---------------------
XP_023900169.1 -------NNKLEKLFPSSDTIZ--------------------------------------------------------------------
XP_023914113.1 -------SEWSDEIL-FGKEAANKGDGCALEGQCMCSEDRHCSPENGYFCKQGLVYKEARVCRYSQSLSGNEYFYGIRYZ----------
XP_043131130.1 -------NDFLVQQLGITRCSGYLSMZ---------------------------------------------------------------
XP_002422445.1 ----------------LSGKVWAERNGILLDZ----------------------------------------------------------
NP_013624.1    INGIIDPSELSDZ-----------------------------------------------------------------------------
XP_003651626.1 -------GPWHRRYLLGERSSAGGEGGEMERLPLEEVGFFTREPAKGGZ-----------------------------------------
XP_049145240.1 -----------KSVVGEAIGVYZ-------------------------------------------------------------------
NP_055577.1    -------PEVYDKICKAARHZ---------------------------------------------------------------------
XP_004926865.1 -------PEIYDKVNRNVRKZ---------------------------------------------------------------------
```

Figure S1
